# Identifizierung und Priorisierung von Forschungsfragen zu Paraplegie infolge einer unfallbedingten Querschnittlähmung mit Betroffenen, Angehörigen und professionell Versorgenden

**DOI:** 10.1055/a-1829-6781

**Published:** 2022-09-09

**Authors:** Michael Levelink, Mona Voigt-Barbarowicz, Carlotta Ahlers, Anna Levke Brütt

**Affiliations:** 1 Department für Versorgungsforschung, Carl von Ossietzky Universitat Oldenburg, Oldenburg, Germany; 2 Forschungspartnerin

**Keywords:** Partizipative Forschung, Querschnittlähmung, James Lind Alliance, Patientenorientierung, Forschungsprioritäten, participatory research, spinal cord injury, James Lind Alliance, patient orientation, research priorities

## Abstract

**Ziel der Arbeit** Menschen mit Paraplegie, Angehörige und professionell Versorgende können mit ihrer Erfahrung und praktischen Kenntnissen zur Entwicklung patientenorientierter, versorgungsrelevanter Forschungsfragen beitragen. Um sie in die Entwicklung einer Forschungsagenda einzubeziehen, hat die James Lind Alliance (JLA) einen etablierten Ansatz entwickelt. Das Ziel dieser Studie ist die Entwicklung einer Forschungsagenda zu Paraplegie infolge einer traumatischen Querschnittlähmung, in Anlehnung an diesen Ansatz.

**Methodik** Es wurden vier aufeinander aufbauende Online-Befragungen unter Menschen mit einer traumatisch bedingten Paraplegie, ihren Angehörigen und professionell Versorgenden durchgeführt. In der ersten haben die Teilnehmenden aus ihrer Sicht unbeantwortete Fragen frei formuliert. Diese wurden zusammengefasst und dahingehend geprüft, ob sie bereits durch Forschung beantwortbar sind. Die unbeantworteten Fragen wurden in weiteren Befragungen schrittweise priorisiert: In der zweiten wurden sie auf einer fünfstufigen Rating-Skala (1–5) hinsichtlich ihrer Relevanz bewertet, um eine Shortlist aus den Fragen zu entwickeln, die mit einem Mittelwert von über 4 bewertet wurden. In der dritten Umfrage wurden daraus die Top 10 bestimmt, deren Rangordnung in der vierten Befragung ermittelt wurde.

**Ergebnisse** Es wurden 38 unbeantwortete Forschungsfragen identifiziert (1. Befragung; n=52). Davon wurden 26 Fragen als wichtig bewertet (2. Befragung; n=53), aus denen 10 Fragen ausgewählt (3. Befragung; n=17) und in eine Rangordnung gebracht wurden (4. Befragung; n=12). Vier Prioritäten betreffen Behandlungsmöglichkeiten der Querschnittlähmung oder damit verbundener Gesundheitsprobleme, drei die Gestaltung der Gesundheitsversorgung im Bereich der Hilfsmittel und der Implementierung von Forschung, zwei adressieren Patientenfaktoren, die zur Verbesserung der eigenen Situation beitragen und eine die Erforschung des Krankheitsverlaufs.

**Schlussfolgerung** Es wurden neun Fragestellungen priorisiert, die auf Forschung zur Verbesserung der Lebens- und Versorgungssituation mit einer Querschnittlähmung abzielen, während eine Frage die Heilung adressiert. Die priorisierten Fragen sollten durch Forschung aufgegriffen werden, damit Problemstellungen adressiert werden, die für Betroffene, Angehörige und professionell Versorgende relevant sind.

## Abstract (en)

**Background** Objective With expertise based on experience, paraplegics, their relatives and health care professionals can contribute to the development of research questions relevant for those affected and those in health care practice. For this purpose, the James Lind Alliance (JLA) has provided a methodological approach. The aim of this study was to develop a research agenda for paraplegia resulting from traumatic spinal cord injury with an adapted JLA approach.

**Methods** Four consecutive online surveys of people with paraplegia caused by traumatic spinal cord injury, their relatives and caregivers were conducted. In the first survey, the respondents freely formulated research questions unanswered from their point of view. These were synthesized and checked to see if they can already be answered by available evidence. The unresolved questions were prioritized stepwise in the subsequent surveys. In the second survey, the relevance of questions was rated on a five-point rating scale (1–5). Questions with a mean value of 4 or higher were taken up in the third survey, in which the 10 most relevant questions were determined. These were ranked in the fourth survey as a top-10 list.

**Results** Based on the first survey (n=52), 38 unresolved research questions were identified. Of these, 26 questions were rated as important (2nd survey; n=53), from which 10 questions were selected (3rd survey; n=17) and ranked (4th survey; n=12) as a top-10 list. Four prioritized questions related to treatment of spinal cord injury or associated health issues, three to aspects of the health care system with regard to assistive devices and the implementation of new therapies, two to possibilities of those affected to improve their own situation, and one to research on the course of disease.

**Conclusions** Nine priorities focus on research that could help improve the life and health care situation of paraplegic patients and one on curative treatment. The prioritized questions should be taken up by researchers and research funders for the benefit of patients and to help health care providers. For some priorities, a need for research was consistently identified in present guidelines or systematic reviews.

## Einleitung

Eine Querschnittlähmung führt zu schwerwiegenden und komplexen Funktionsbeeinträchtigungen, die sich erheblich auf das alltägliche Leben auswirken. Die Versorgungs- und Lebenssituation Betroffener in Deutschland ist bisher kaum erforscht 1 . Konkretere Einblicke lieferte zuletzt die International Spinal Cord Injury (InSCI) Survey 2 , eine internationale Kohortenstudie an der 12 591 Personen mit Querschnittlähmung aus 22 Ländern teilgenommen haben 3 . In den deutschen Studienarm wurden 1.479 Personen eingeschlossen. Davon lag bei 74,3% eine traumatische Ursache und bei 51,2% eine Paraplegie vor 4 . Schätzungen zur bundesweiten Inzidenz gehen von 2200 Neuerkrankungen pro Jahr aus, von denen die Hälfte traumatisch bedingt ist 1 .

Gesundheitliche Problemlagen, die mit einer Querschnittlähmung einhergehen, soziodemografische Charakteristika 5 6 und die Inanspruchnahme von Versorgungsleistungen 7 unterscheiden sich zwischen Betroffenen mit traumatischer oder nicht-traumatischer Ätiologie, sodass diese nicht als homogene Gruppe in der Forschung betrachtet werden können. 5 6 . Auch die Lebenssituation von Tetra- und Paraplegiker*innen unterscheidet sich deutlich. Tetraplegiker*innen berichten in nahezu allen Körperfunktions-, Aktivitäts- und Teilhabebereichen signifikant häufiger Probleme 6 .

Um sicherzustellen, dass Forschung patienten- und versorgungsrelevante Fragestellungen verfolgt und die Träger*innen der Krankheits- und Behandlungsbelastung über Forschung mitentscheiden zu lassen 8 , werden Betroffene und professionell Versorgende zunehmend als Expert*innen mit Behandlungs- und Krankheitserfahrungen in Forschung einbezogen 9 . Dies kann in verschiedenen Phasen des Forschungsprozesses erfolgen, in dem die Bestimmung des Forschungsbedarfs eine erste zentrale Entscheidung darstellt 10 . Damit Betroffene, Angehörige und professionell Versorgende in diesen Schritt stärker einbezogen werden, wurde 2004 in Großbritannien die James Lind Alliance (JLA) gegründet. Dabei handelt es sich um eine staatlich unterstützte Initiative, im Rahmen derer die Methodik der Priority Setting Partnerships (PSP) entwickelt und umgesetzt wird. Diese sieht vor, dass jeweils zu spezifischen Gesundheitsproblemen oder Versorgungssettings eine PSP durchgeführt wird, unter Leitung eines Projektteams, in dem Betroffene, Angehörige und professionell Versorgende vertreten sind. Im Rahmen einer PSP wird zunächst eine Umfrage durchgeführt, in der weitere Betroffene, Angehörige und professionell Versorgende aus ihrer Sicht unbeantwortete Fragen mitteilen. Diese werden von dem Projektteam zusammengefasst und es wird geprüft, welche Fragen bereits durch Forschung beantwortet wurden. Die unbeantworteten Fragen werden anschließend durch Betroffene, Angehörige und professionell Versorgende priorisiert, um eine Top 10 Liste als patientenorientierte Forschungsagenda zu dem jeweiligen Gesundheitsproblem oder Versorgungssetting zu entwickeln 11 . Dieser Ansatz wurde bereits in zahlreichen PSPs zu verschiedenen Gesundheitsproblemen eingesetzt. Neben dem JLA-Ansatz werden verschiedene weitere Methoden wie Gruppendiskussionen, Interviews, Umfragen oder Workshops kombiniert, um eine Forschungsagenda mit Patienten zu entwickeln 12 . Eine Auswertung der ersten 14 in Großbritannien durchgeführten JLA-PSPs zeigt, dass sich die darin priorisierten Themenschwerpunkte von denen registrierter Studien unterscheiden. Während darin prioritär zu medikamentösen Therapien geforscht wird, wurden mit dem JLA-Ansatz vor allem Forschungsfragen zu nicht-medikamentösen Behandlungsmöglichkeiten priorisiert (z. B. Patientenedukation, Physiotherapie) 13 .

In Großbritannien wurde bereits eine PSP zu Rückenmarksverletzungen durchgeführt, in die Betroffene mit traumatischer und nicht-traumatischer Querschnittlähmung sowie mit Para- und Tetraplegie eingeschlossen wurden 14 . Die Forschungsprioritäten dieser Gruppen können aufgrund der unterschiedlichen Problemlagen jedoch voneinander abweichen. Zudem können sich die Forschungsprioritäten in unterschiedlichen Versorgungskontexten unterscheiden, sodass in Großbritannien entwickelte Fragen nur bedingt auf Deutschland übertragbar sind. Das Ziel der vorliegenden Studie besteht daher in der Entwicklung einer Forschungsagenda zur traumatisch bedingten Paraplegie mit Betroffenen, Angehörigen und professionell Versorgenden aus dem deutschen Versorgungskontext. Als Ergebnis soll eine Top 10 Liste von Forschungsprioritäten zur traumatisch bedingten Querschnittlähmung entwickelt werden, die Forschenden und Forschungsfördernden als Orientierung dienen kann, um ihre Arbeit näher an den Interessen derer auszurichten, die an der alltäglichen Versorgung beteiligt sind.

## Methodik

Die Beschreibung der Methodik orientiert sich an der vorläufigen Version der Reporting guideline for priority setting of health research (REPRISE), die spezifisch für Priorisierungsstudien entwickelt wurde (Anhang A) 15 16 .

**Abb. 1:**
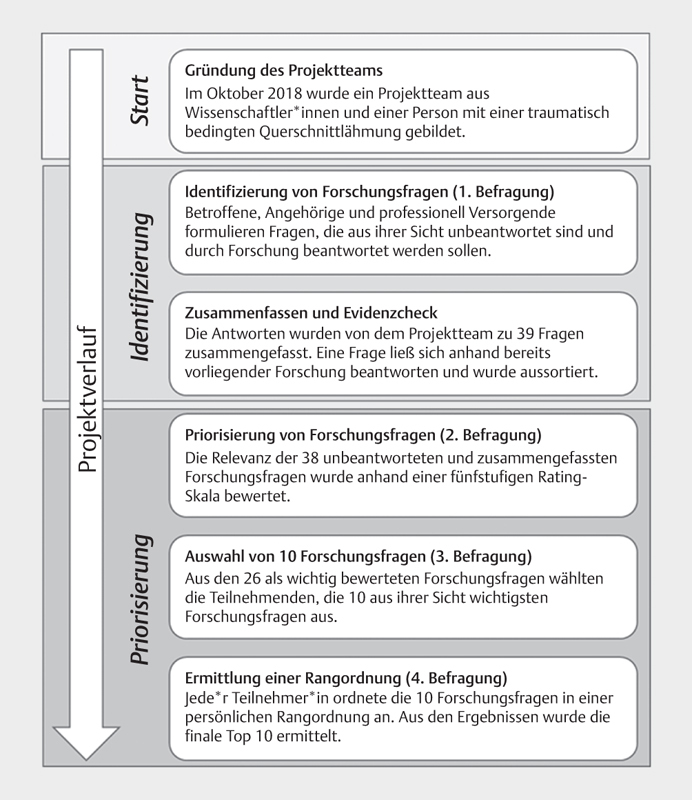
Ablauf der Studie.

Das Studiendesign wurde in Anlehnung an die Empfehlungen der JLA konzipiert 11 . Das Projektteam bestand aus drei Wissenschaftler*innen (ML, MVB, ALB) sowie einer Person mit traumatisch bedingter Paraplegie (CA). Eine der Wissenschaftler*innen verfügte bereits über Erfahrungen in der Durchführung von Priorisierungsstudien. Es wurden vier aufeinander aufbauende Online-Befragungen durchgeführt. Die erste diente der Identifizierung von Forschungsfragen, die in drei weiteren Befragungen priorisiert wurden ( Abb. 1 ). Die Forschungsfragen wurden nicht auf bestimmte wissenschaftliche Disziplinen eingegrenzt.

### Teilnehmer*innen

In die Studie wurden deutschsprachige Personen eingeschlossen, die von einer traumatisch bedingten Querschnittslähmung betroffen sind, Angehörige (Lebenspartner*innen, Familie, Freunde) sowie professionell Versorgende (Ärzt*innen, Pflegekräfte, Therapeut*innen). Um Teilnehmer*innen für die ersten beiden Umfragen zu rekrutieren, wurden jeweils 38 Akut- und Rehakliniken aus Deutschland mit paraplegiologischem Behandlungsangebot sowie Informationsportale und Interessensvertretungen zu Querschnittlähmung kontaktiert und gebeten, Informationen zur Studienteilnahme an die Zielgruppe weiterzuleiten. Zudem wurde die Umfrage über Gruppen zu Querschnittlähmung in sozialen Medien verbreitet. Zur dritten und vierten Umfrage wurden Teilnehmer*innen der ersten beiden Befragungen eingeladen, die dafür ihre E-Mailadresse angaben. Die Teilnahme war freiwillig und wurde nicht vergütet.

### Identifizierung von Forschungsfragen

Die erste Online-Umfrage zur Identifizierung von Forschungsfragen wurde von Anfang Dezember 2018 bis Ende März 2019 durchgeführt. Darin wurden die Teilnehmer*innen in einem offenen Format aufgefordert, aus ihrer Sicht unbeantwortete Fragen zur Querschnittlähmung mitzuteilen. Zudem wurden, wie auch in den Folgebefragungen, soziodemografische Charakteristika abgefragt und welcher Bezug zur Querschnittlähmung besteht (Betroffene; Angehörige; professionell Versorgende).

### Zusammenfassung und Evidenzprüfung

Entsprechend der JLA-Empfehlungen 11 wurden die Fragen von zwei Mitgliedern des Projektteams (ML, MVB) unabhängig voneinander in induktiv gebildete Kategorien eingeordnet. Beiträge, die mehrere Forschungsfragen enthielten, wurden mehreren Kategorien zugeordnet. Basierend auf dieser Einordnung haben alle Autor*innen anschließend konsensual finale Kategorien festgelegt, über die Zuordnung jeder Frage zu diesen entschieden und auf dieser Basis die Fragen zusammengefasst. Fragen wurden aussortiert, wenn alle Autor*innen konsensual entschieden haben, dass es sich um nicht durch Forschung beantwortbare Informationsbedarfe handelt (z. B. „Wie weit ist die Forschung bezüglich der Heilung einer Querschnittslähmung?“).

Im Juli 2019 wurde überprüft, ob sich die zusammengefassten Forschungsfragen bereits beantworten lassen. Dazu wurde jede Frage von zwei Mitgliedern des Projektteams unabhängig voneinander zu einer Suchstrategie operationalisiert, anhand derer in PubMed nach maximal 10 Jahre alten systematischen Reviews gesucht wurde. Anschließend wurde im gesamten Projektteam diskutiert und konsensual entschieden, inwieweit eine Frage aufgrund der jeweils gefundenen Reviews als beantwortet angesehen werden kann. Bereits beantwortete Fragen wurden von der weiteren Priorisierung ausgeschlossen.

### Priorisierung der Forschungsfragen

Die zusammengefassten, unbeantworteten Fragestellungen wurden in einer zweiten Umfrage von Mitte April 2020 bis Ende Juli 2020 hinsichtlich Ihrer Relevanz bewertet. Darin beurteilten die Teilnehmer*innen auf einer Rating-Skala von 1 (gar nicht) bis 5 (außerordentlich), wie wichtig es ihnen ist, dass Forschung diese Frage aufgreift. Aus den Fragen mit einem Mittelwert (MW) von 4 oder höher wurde eine Shortlist erstellt. Daraus haben die Teilnehmer*innen der dritten Umfrage (Mitte Dezember 2020 bis Mitte Januar 2021) 10 Fragen ausgewählt, die aus ihrer Sicht am wichtigsten sind. Unter den 10 am häufigsten gewählten Fragen wurde in der vierten Umfrage (Mitte bis Ende Januar 2021) eine Rangordnung bestimmt. Dazu haben die Teilnehmenden die 10 Fragen in eine persönliche Rangordnung sortiert. Zur Bestimmung der finalen Top 10 wurden die Fragen nach den zugeordneten Rängen bepunktet (1.Platz=10 Punkte…10.Platz=1 Punkt). Die Teilnehmenden gaben zudem auf einer fünfstufigen Rating-Skala an, inwieweit sie der Auswahl der finalen Fragen zustimmen.

Um prioritäre Themenbereiche zu identifizieren, wurden die Forschungsfragen schließlich von ML deduktiv Kategorien zugeordnet, die in einem systematischen Review aus 34 Priorisierungsstudien entwickelt wurden 12 .

## Ergebnisse

In der ***Identifizierung*** formulierten 52 Teilnehmende aus Ihrer Sicht relevante Forschungsfragen. Darunter waren 24 Betroffene, 4 Angehörige, 21 professionell Versorgende und 4 professionell Versorgende mit einer Querschnittlähmung ( Tab. 1 ). Die Beiträge wurden von dem Projektteam zu 39 Forschungsfragen ***zusammengefasst*** , von denen eine sich anhand eines systematischen Reviews 17 beantworten ließ und ausgeschlossen wurde („Welche Resilienzfaktoren helfen Querschnittsgelähmten bei der psychischen Verarbeitung?“). Die 38 verbleibenden Forschungsfragen adressieren ein breites inhaltliches Spektrum, das alle Kategorien des zugrundeliegenden Review 12 umfasst ( Tab. 2 ).

**Table:** **Tab. 1** Zusammensetzung der Stichproben.

	Identifizierung		Priorisierung					
	1. Befragung		2. Befragung		3. Befragung		4. Befragung	
**Geschlecht**	**n**	**%**	**n**	**%**	**n**	**%**	**n**	**%**
Männlich	26	50%	30	57%	9	53%	8	67%
Weiblich	26	50%	23	43%	8	47%	4	33%
**Alter in Jahren**								
Mittelwert	46		46		48		50	
Minimum-Maximum	18–64		20–68		30–64		30–64	
**Bezug zum Thema**	**n**	**%**	**n**	**%**	**n**	**%**	**n**	**%**
selbst betroffen	28	54%	36	68%	11	65%	8	67%
Angehörige	4	8%	3	6%	1	6%	0	
professionell Versorgende	25	48%	14	26%	7	41%	4	33%
**Gesamt**	**52**		**53**		**17**		**12**	

*Mittelwert und Median der Punkte aus der vierten Befragung

Die 38 unbeantworteten Fragen wurden in der zweiten Befragung von 53 Teilnehmer*innen ***priorisiert*** ( Tab. 1 ; Anhang B). Dabei wurden 26 Fragen mit einem Durchschnittswert von über 4 bewertet. Daraus haben 17 Teilnehmende in der dritten Befragung 10 Fragen bestimmt, aus denen in der vierten Befragung von 12 Teilnehmenden die finale Top 10 gebildet wurde ( Tab. 2 ). Sie stimmten der Auswahl von Forschungsprioritäten mittelmäßig (n=3), ziemlich (n=8) oder sehr (n=1) zu (Median=4; MW=3,83).

**Table:** **Tab. 2** Top 10 der Forschungsprioritäten zu traumatisch bedingter Querschnittlähmung.

Rang	Forschungsfrage	Kategorien nach 12	MW*	Med*
**1**	Wie können chronische Schmerzen effektiver behandelt werden?	Behandlung	6,75	8,50
**2a**	Wie können Forschungsergebnisse und Therapieformen besser in der Versorgung umgesetzt werden?	Versorgungssystem	5,83	6,50
**2b**	Wie können Betroffene besser dabei gefördert werden, Expert*innen der eigenen Querschnittlähmung zu werden?	Patientenfaktoren	5,83	6,00
**4**	Wie kann der Hilfsmittelversorgungsprozess verbessert werden?	Versorgungssystem	5,67	5,00
**5**	Können (neue) Hilfsmittel und technische Lösungen das Blasen- und Darmmanagement verbessern?	Behandlung	5,50	5,50
**6a**	Was sind die Langzeitfolgen einer Querschnittlähmung?	Gesundheitsproblem	5,42	6,00
**6b**	Können sich verletzte Nerven (z. B. durch Elektrostimulation, Tissue Engineering) wieder regenerieren?	Behandlung	5,42	5,00
**8**	Welchen Einfluss hat die Qualität eines Hilfsmittels (z. B. Rollstuhl) auf gesundheitsbezogene und ökonomische Outcomes?	Versorgungssystem	5,17	5,50
**9**	Welche physiotherapeutischen Behandlungen sind effektiv?	Behandlung	5,00	4,50
**10**	Welchen Einfluss hat körperliche Aktivität auf den Gesundheitszustand?	Patientenfaktoren	4,42	3,50

^*^ Mittelwert und Median der Punkte aus der vierten Befragung.

## Diskussion

Dies ist unseres Wissens die erste Priorisierungsstudie zu Paraplegie infolge einer traumatisch bedingten Querschnittlähmung und die erste zu Querschnittlähmungen aus dem deutschen Versorgungskontext. Von den priorisierten Fragen richtet sich eine auf die kurative Behandlung der Rückenmarksverletzung ( ***#6b*** ), während die anderen auf Forschung zur Verbesserung der Lebensqualität und Versorgungssituation mit einer Querschnittlähmung abzielen. Anhand der zugeordneten Kategorien 12 zeigt sich, dass vier Forschungsfragen zu Behandlungsmöglichkeiten für das Gesundheitsproblem oder Komorbiditäten, drei zum Versorgungssystem, zwei zu Patientenfaktoren und eine zum Gesundheitsproblem priorisiert wurden. Damit sind in der vorliegenden Priorisierungsliste die vier Themengebiete vorzufinden, die auch in Priorisierungsstudien zu anderen Gesundheitsproblemen am häufigsten priorisiert wurden, in denen der JLA-Ansatz adaptiert wurde. Der Schwerpunkt der Priorisierungsstudie zu Rückenmarkverletzungen aus Großbritannien 14 liegt dagegen stärker auf Forschung zu Behandlungsmöglichkeiten, wie auch in anderen von der JLA durchgeführten Priorisierungsstudien 12 . Insbesondere an den Forschungsfragen zur Kategorie Versorgungssystem zeigt sich die Kontextspezifität einer Forschungsagenda. Entsprechende Fragen wurden in der britischen PSP nicht priorisiert. Inhaltliche Überschneidungen zu dieser PSP finden sich zum Beispiel bei Forschungsfragen zu Regenerationspotenzialen durch Elektrostimulation oder Langzeitfolgen einer Querschnittlähmung 14 .

**Einordnung der zusammengefassten Forschungsfragen in die Kategorien nach [13].**

**Behandlung** Kann die Blasenfunktion durch frühes Training erhalten bleiben? Kann eine Querschnittlähmung durch die Beeinflussung von Bewusstseinszuständen (z. B. Meditation, Hypnose) geheilt werden? Können (neue) Hilfsmittel und technische Lösungen das Blasen- und Darmmanagement verbessern? Können sich verletzte Nerven (z. B. durch Elektrostimulation, Tissue Engineering) wieder regenerieren? Sind Kompressionsstrümpfe zur Thromboseprophylaxe wirksam? Welche physiotherapeutischen Behandlungen sind effektiv? Welchen Einfluss haben Medikamente auf das Blasenmanagement? Wie kann chronischen Schmerzen besser vorgebeugt werden? Wie kann Osteoporose behandelt werden? Wie können Betroffene mit psychischen Krisen (z. B. Suizidalität, Sucht) besser behandelt werden? Wie können chronische Schmerzen effektiver behandelt werden? Wie können Harnwegsinfekte besser behandelt werden? Wie können Spastiken effektiver behandelt werden?

**Gesundheitsversorgungssystem** Welche Faktoren (z. B. Dauer, Rehabilitationsverständnis) haben Einfluss auf die Qualität der rehabilitativen Versorgung von Menschen mit Querschnittlähmung? Welchen Einfluss hat die Qualität eines Hilfsmittels (z. B. Rollstuhl) auf gesundheitsbezogene und ökonomische Outcomes? Wie ist die Qualität der ambulanten Versorgung von Menschen mit Querschnittlähmung einzuschätzen? Wie kann der Hilfsmittelversorgungsprozess verbessert werden? Wie kann die Gewährung von Sozialleistungen ökonomischer gestaltet werden? Wie kann ich als forschungsinteressierte * r Behandler * in, Angehörige * r und Betroffene * r besser an Studien mitarbeiten? Wie können Forschungsergebnisse und Therapieformen besser in der Versorgung umgesetzt werden? Wie können Menschen mit Querschnittslähmung besser mit Psychotherapie versorgt werden?

**Patientenfaktoren** Können Ernährungsempfehlungen das Darmmanagement der Betroffenen verbessern? Können Menschen mit Querschnittslähmung gesellschaftlich teilhaben und ihr Leben selbständig gestalten? Welchen Einfluss hat körperliche Aktivität auf den Gesundheitszustand? Wie entwickelt sich das psychische Befinden nach der Erstrehabilitation? Wie kann der Bewältigungsprozess bei Betroffenen besser unterstützt werden? Wie können Betroffene besser dabei gefördert werden, Expert*innen der eigenen Querschnittlähmung zu werden?

**Gesundheitsproblem** Was sind die Langzeitfolgen einer Querschnittlähmung? Welche Risikofaktoren für Dekubitus gibt es? Welchen Einfluss hat der Monatszyklus auf das Blasenmanagement?

**Professionell Versorgende** Welche Arbeitsbedingungen müssen geboten werden, um die Arbeit für Pflegende in der Paraplegiologie attraktiv zu machen? Welchen Einfluss haben Ausbildungsaspekte (z. B. Kommunikationstrainings) von Behandelnden auf ihren Umgang mit Menschen mit Querschnittlähmung? Wie sind die psychosozialen Kompetenzen von den an der Versorgung beteiligten Berufsgruppen ausgeprägt?

**Prävention** Wie kann Dekubitus besser vorgebeugt werden? Wie kann Osteoporose vorgebeugt werden?

**Diagnose** Wie können Harnwegsinfekte besser diagnostiziert werden?

**Nichtprofessionelle Versorgende** Wie können Angehörige besser unterstützt werden?

**Öffentlichkeitsarbeit** Wie kann die Öffentlichkeit besser über Querschnittlähmung und ihre Folgen aufgeklärt werden?

Um Ansatzpunkte für anknüpfende Forschung aufzuzeigen, werden die priorisierten Forschungsfragen im Folgenden jeweils anhand vorliegender Literatur diskutiert:

Die drei Fragen zur Behandlung adressieren mit Schmerztherapie ( ***#1*** ), Physiotherapie ( ***#9*** ) sowie Blasen- und Darmmanagement ( ***#5*** ) gesundheitliche Problemlagen, die mit einer Querschnittlähmung einhergehen können. Vorliegende Leitlinien zur Querschnittlähmung weisen ebenfalls auf diesbezügliche Forschungsbedarfe hin. Es lägen beispielsweise nur wenige Primärdaten zur Schmerztherapie bei einer Querschnittlähmung 18 vor, sowie zu evidenzbasierten physiotherapeutischen Behandlungsmethoden 19 . In diesen Prioritäten finden sich auch die Problemlagen wieder, die in der deutschen InSCI-Teilstudie als am zweit bis sechsthäufigsten als schwerwiegend beschrieben wurden (Gelenk- und Muskelschmerz, Schmerz, Kontrakturen, Muskuläre Spastik, Harnblasenfunktionsstörungen, Darmfunktionsstörungen). Zu dem häufigsten schwerwiegenden Problem, Störungen der Sexualität 4 , wurde dagegen keine Forschungsfrage entwickelt. Das könnte darin begründet sein, dass sensible Themen auch in anonymen Umfragen weniger berichtet werden 20 oder darin, dass dieses persönliche Problem weniger als wenig zugänglich für Forschung eingeschätzt wird. Auch in den Top 10 der JLA PSP zu Querschnittlähmungen aus Großbritannien taucht das Thema nicht auf 14 . Eine priorisierte Forschungsfrage thematisiert die Heilung verletzter Nerven ( ***#6b*** ). Derzeit wird an verschiedenen neuroregenerativen Ansätzen geforscht, zum Beispiel auf Basis von Medikamenten, Neurostimulation oder Stammzelltherapie. Diese werden in klinischen und präklinischen Studien erprobt, in der Versorgung aber noch nicht eingesetzt 21 .

Zwei Fragen zum Versorgungssystem adressieren die Hilfsmittelversorgung ( ***#4, #8*** ). Darin spiegelt sich die zentrale Bedeutung von Hilfsmitteln im Leben mit Querschnittlähmung wider 22 sowie Befunde aus der InSCI-Studie, die für Deutschland und die Schweiz deutlichen Verbesserungsbedarf in der Hilfsmittelversorgung aufzeigen 23 . Ein Vergleich mit Hilfsmittelversorgungskonzepten aus Ländern, in denen die Hilfsmittelversorgung weniger problematisch erlebt wird (z. B. Norwegen, Japan) 23 , könnte Ansatzpunkte zur Verbesserung der Hilfsmittelversorgung aufdecken. Die dritte Frage zum Versorgungssystem betrifft eine möglichst zügige Implementierung von Forschung in die Versorgung ( ***#2a*** ). Dieses Interesse könnte durch das Potenzial neuroregenerativer Ansätze begründet sein.

Eine Forschungsfrage zu Patientenfaktoren fragt nach dem Einfluss körperlicher Aktivität auf den Gesundheitszustand ( ***#10*** ). Teilaspekte dieser Frage wurden bereits untersucht, jedoch wird in entsprechender Literatur auch auf einen Mangel an Forschungsarbeiten hingewiesen 24 . Die zweite Frage zu Patientenfaktoren adressiert bessere Möglichkeiten, um Betroffene zu Expert*innen der eigenen Querschnittlähmung auszubilden ( ***#2b*** ). Ein Scoping-Review mit 112 eingeschlossenen Artikeln zeigt, dass bereits zahlreiche Selbstmanagement-Interventionen zu Querschnittlähmungen entwickelt und erprobt wurden, aber auch, dass es an vergleichenden Untersuchungen mangelt, die am besten geeigneten Ansätze identifizieren 25 .

Eine priorisierte Frage adressiert Langzeitfolgen einer Querschnittlähmung ( ***#6a*** ), zu der aus dem längsschnittigen Ansatz der InSCI Survey weitere Erkenntnisse hervorgehen könnten 2 . Die Langzeitfolgen können vielfältig sein und wurden bereits teilweise in neurologischer 26 oder psychosozialer Hinsicht untersucht 27 .

Die Gegenüberstellung zur aktuellen Forschungslage zeigt, dass einige Fragen bereits aufgegriffen werden. Es werden aber auch weitere Forschungsbedarfe aufgezeigt, zur Evidenz von unterschiedlichen Behandlungsansätze und Selbstmanagement-Interventionen oder auch zur Optimierung der Hilfsmittelversorgung.

In partizipativer Versorgungsforschung sollen Betroffene von der Identifizierung des Forschungsbedarfs bis hin zur Dissemination der Ergebnisse in den Forschungsprozess einbezogen werden 28 . Mit dieser Studie haben wir nun Informationen zum Forschungsbedarf aus Sicht derer bereitgestellt, die an der alltäglichen Versorgung nach einer unfallbedingten Querschnittlähmung beteiligt sind. Auch in Forschung, die daran anknüpft, sollten sie als Expert*innen zu alltags- und versorgungsrelevanten Aspekten einbezogen werden, damit ihre Perspektiven in Ausdifferenzierung und Untersuchung der Fragestellungen Berücksichtigung finden.

### Limitationen

Die Befragungen wurden als Online-Umfragen durchgeführt. Da keine personenbezogenen Daten mit den Antworten verknüpft wurden, ist nicht überprüfbar, inwieweit sich Teilnehmer*innen an mehreren Befragungen beteiligt haben oder ob Personen außerhalb der Zielgruppe teilgenommen haben. Außerdem könnte das Online-Format Personen ausgeschlossen haben. Um sicherzustellen, dass die Befragung zugänglich ist und die Zielgruppe erreicht wird, wurde eine Person mit unfallbedingter Querschnittlähmung in den Pretest und die Rekrutierung eingebunden.

Diese war auch in die Zusammenfassung der Forschungsfragen, in den Evidenzcheck und die Dissemination der Ergebnisse eingebunden. Um als Ko-Forschende an solchen Arbeitsschritten partizipieren können, wird grundlegendes Vorwissen zum wissenschaftlichen Arbeiten benötigt, zum Beispiel zur Logik qualitativer Forschung oder zum Konzept der Evidenz. Eine Stärke unserer Studie besteht darin, dass die Betroffene aus unserem Projektteam zum Projektstart über mehrere Monate in Vollzeit an der Arbeitsgruppe beschäftigt war. In dieser Zeit eignete sie sich grundlegende forschungsbezogene Kompetenzen an. Sind entsprechende Voraussetzungen nicht gegeben, kann ein zielgruppenspezifisches Forschungstraining bei der Ausbildung forschungsbezogener Kompetenzen unterstützen. Ebenso bedarf es jedoch auf Seiten der Wissenschaftler*innen einer Auseinandersetzung mit methodischen Überlegungen und mit der eigenen Einstellung zu partizipativer Forschung 29 .

Für die Studie konnten relativ wenige Angehörige rekrutiert werden. Bei einer höheren Beteiligungsquote wären möglicherweise mehr Forschungsfragen zu deren Problemlagen priorisiert worden. Eine entsprechende Fragestellung wurde identifiziert („Wie können Angehörige besser unterstützt werden?“), ist jedoch in der dritten Befragung ausgeschieden.

Eine Stärke der Studie besteht in der Nutzung des etablierten methodischen Ansatzes der JLA. Bei der Festlegung der finalen Top 10 wurde jedoch von den JLA-Empfehlungen abgewichen, die einen persönlichen Workshop vorsehen 11 . Für die Online-Umsetzung wurde die Top 10 über zwei Umfragen bestimmt. In der letzten Umfrage haben keine Teilnehmenden der Themenauswahl nicht oder nur wenig zugestimmt, sodass die Prioritätenliste als ein Konsens gesehen werden kann, dem alle mittel bis sehr zustimmten. Jedoch ermöglichte das Online-Verfahren anders als in einem Workshop nicht den Austausch und die Diskussion unterschiedlicher Perspektiven 11 .

### Schlussfolgerung

Betroffene, Angehörige und professionell Versorgende entwickeln aus ihrer Situation Forschungsfragen zu spezifischen Versorgungs- und Lebenssituationen. Durch die Berücksichtigung dieser Fragen bei der Konzeption von Forschungsprojekten und Förderprogrammen kann Forschung stärker auf die Interessen von Betroffenen und praktisch relevante Fragestellungen ausgerichtet werden. Wenngleich einige priorisierte Fragen bereits in laufenden Forschungsarbeiten adressiert werden, bestehen weitere Forschungsbedarfe. Im nächsten Schritt bedarf es des Engagements von Forschenden in unterschiedlichen Disziplinen, damit die vorliegende Priorisierungsstudie zu einer höheren Relevanz von Forschung für Betroffene, Angehörige und professionell Versorgende beitragen kann 30 . Ebenso bedarf es veränderter Förder- und Rahmenbedingungen für Gesundheitsforschung, die wirksame Anreize für eine qualitativ hochwertige Umsetzung von Partizipation darstellen 31 .

## Zusätzliches Material

Zusätzliches Material Zusätzliches Material
