# Supplementary material for: Identification and Prioritization of Research Questions on Paraplegia Caused by Traumatic Spinal Cord Injury with Those Affected, Their Relatives and Health Care Professionals
Source: Gesundheitswesen. 2022 Sep 9;85(4):250–7. [Article in German] doi: 10.1055/a-1829-6781 (PMC10125339; doi:10.1055/a-1829-6781)
Supplement: Supplementary file 1 — Zusätzliches Material [file 10-1055-a-1829-6781-2021-09-1515.pdf]

Zusatzmaterial

Anhang

A. Checkliste der Reporting guideline for priority setting of health research (REPRISE)

B. Antworten aus allen vier Befragungsrunden

Anhang A. Checkliste der Reporting guideline for priority setting of health research (REPRISE)

No	Item	Descriptor and/or *examples*	Page
**A**	**Context and scope**		
1	Define geographical scope	Global, regional, national, city, local area, institutional/organisational level, health service	3
2	Define health area, field, focus	Disease or condition specific, interventions, healthcare delivery, health system	3
3	Define end-users of research	Intended beneficiaries e.g. general population or a specific population based on demographic (age, gender), clinical (disease, condition), or other characteristics	7
4	Define the target audience of the priorities	Policy makers, funders, researchers, industry	3, 7
5	Identify the broad research area	Public health, health services research, clinical research, basic science	3, 5
6	Identify the type of research question	Etiology, diagnosis, prevention, treatment (interventions), prognosis, health services, psychosocial, behavioral and social science, economic evaluation, implementation	5
7	Define the time frame	Interim, short-term, long-term priorities, plans to revise and update	
**B**	**Governance and team**		
8	Describe selection of the leadership and management team	Those responsible for initiating, developing, and guiding the process for priority setting e.g. Steering Committee, Advisory Group, Technical Experts	3
9	Describe the characteristics of the team, and the networks they represent	Stakeholder groups, organisations or networks represented, characteristics (demographics, experience, expertise)	3
10	Describe any training or experience in priority setting	Consultants or advisors with experience in priority setting	3,6
**C**	**Framework for priority setting**		
11	State the framework used (if any)	James Lind Alliance, COHRED, CHNRI, no framework	2-3
**D**	**Stakeholders or participants**		
12	Define the inclusion criteria for stakeholders involved in priority-setting	Patients, caregivers, general community, health professionals, researchers, policy makers, non-governmental organisations, government, industry; specific groups including vulnerable and marginalized populations	3
13	State the strategy or method for identifying and engaging stakeholders	Partnership with organizations, social media, recruitment through hospitals	3
14	Indicate the number of participants and/or organisations involved	Number of individuals and organisations, include number by stakeholder group	4
15	Describe the characteristics of stakeholders	Stakeholder group, demographic characteristics, areas of interest and expertise, discipline, affiliations	4
16	State if reimbursement for participation was provided	Cash, vouchers, certificates, acknowledgement; what purpose e.g. travel, accommodation, honorarium	3

**E**	**Identification and collection of research priorities**		
17	Describe methods for collecting priorities from stakeholders	Methods e.g. Delphi survey, surveys, nominal group technique, interviews, focus groups, meetings, workshops; prioritization e.g. voting, ranking; mode e.g. face-to-face, online; may be informed by evidence e.g, systematic reviews, reviews of guidelines/other documents	3
18	Describe methods for collating and categorizing priorities	Taxonomy or other framework used to organise, summarise, and aggregate topics or questions	3-4
19	Describe methods and reasons for removing priorities	Based on scope, clarity, definition, duplication, other criteria	3-4
20	Describe methods for refining or translating priorities into research topics or questions	Reviewed by Steering Committee or project team	3-4
21	Describe methods for checking whether research questions or topics have been answered	Systematic reviews, evidence mapping, consultation with experts	3
22	Describe number of research questions or topics	Number of priorities at each stage of the process	4, Anhang B
**F**	**Prioritisation of research topics/questions**		
23	Describe methods and criteria for prioritising research topics or questions	Methods e.g. Delphi survey, surveys, nominal group technique, interviews, focus groups, meetings, workshops; prioritisation e.g. voting, ranking; mode e.g. face-to-face, online; criteria e.g. need, feasibility, novelty, equity	4
24	Provide reasons for excluding research topics/questions	Thresholds for ranking scores, proportions, votes; other criteria	3
**G**	**Output**		
25	Specificity of research priorities are clear	Area, topic, questions, PICO (population, intervention, comparator, outcome)	
**H**	**Evaluation and feedback**		
26	Describe how the process of prioritization was evaluated	Survey, workshop	4
27	Describe the approach for feeding back priorities to stakeholders and/or to the public; and how feedback was addressed and integrated	Public meetings or workshop, newsletters, website, email	3-4, 7
**I**	**Implementation**		
28	Outline the strategy or action plans for implementing priorities	Communication with target audience, via policies and funding	6-7
29	Describe evaluation of impact	Integration in decision-making, funding allocation	N/A
**J**	**Funding and conflict of interest**		
30	State sources of funding	Name sources of funding for the priority-setting exercise	7
31	Outline the budget and/or cost	Indicate budget and cost	N/A
32	Provide declaration of conflict of interest	Statement of conflict of interest	7

Anhang B. Antworten aus allen vier Befragungsrunden

	Identifizierung			***Priorisierung***		
	**1. Befragung (n=52)**	**Kategorien[13]**	**adressiert von**	**2. Befragung (n=53)**	**3. Befragung (n=17)**	**4. Befragung (n=12)**
#	Identifizierte Forschungsfragen		Gruppen[1]	Mittelwert[2]	Häufigkeit [3]	Punkte[4]
1	Wie können chronische Schmerzen effektiver behandelt werden?	Behandlung	B, V	4,44	8	81
2	Wie können Forschungsergebnisse und Therapieformen besser in der Versorgung umgesetzt werden?	Gesundheitsversorgungssystem	B, V	4,33	8	70
	Wie können Betroffene besser dabei gefördert werden, Expert*innen der eigenen Querschnittlähmung zu werden?	Patientenfaktoren	V	4,20	8	70
4	Wie kann der Hilfsmittelversorgungsprozess verbessert werden?	Gesundheitsversorgungssystem	B, V	4,32	8	68
5	Können (neue) Hilfsmittel und technische Lösungen das Blasen- und Darmmanagement verbessern?	Behandlung	B, A, V	4,49	8	66
6	Können sich verletzte Nerven (z.B. durch Elektrostimulation, Tissue Engineering) wieder regenerieren?	Behandlung	B	4,14	11	65
	Was sind die Langzeitfolgen einer Querschnittlähmung?	Gesundheitsproblem	B	4,57	9	65
8	Welchen Einfluss hat die Qualität eines Hilfsmittels (z.B. Rollstuhl) auf gesundheitsbezogene und ökonomische Outcomes?	Gesundheitsversorgungssystem	B	4,26	9	62
9	Welche physiotherapeutischen Behandlungen sind effektiv?	Behandlung	B	4,26	9	60
10	Welchen Einfluss hat körperliche Aktivität auf den Gesundheitszustand?	Patientenfaktoren	B, V	4,37	8	53
11	Wie können Harnwegsinfekte besser behandelt werden?	Behandlung	B, A, V	4,52	7	
12	Wie kann chronischen Schmerzen besser vorgebeugt werden?	Behandlung	B	4,39	7	
13	Wie ist die Qualität der ambulanten Versorgung von Menschen mit Querschnittlähmung einzuschätzen?	Gesundheitsversorgungssystem	B, V	4,37	7	
14	Wie können Spastiken effektiver behandelt werden?	Behandlung	B, V	4,28	7	
15	Wie können Angehörige besser unterstützt werden?	Nichtprofessionelle Versorgende	B, A, V	4,04	7	
16	Wie kann Dekubitus besser vorgebeugt werden?	Prävention	B, A	4,43	6	
17	Wie kann Osteoporose vorgebeugt werden?	Prävention	A	4,04	6	
18	Wie kann der Bewältigungsprozess bei Betroffenen besser unterstützt werden?	Patientenfaktoren	B, A, V	4,04	6	
19	Können Ernährungsempfehlungen das Darmmanagement der Betroffenen verbessern?	Patientenfaktoren	V	4,26	5	
20	Wie kann die Gewährung von Sozialleistungen ökonomischer gestaltet werden?	Gesundheitsversorgungssystem	B	4,10	5	
21	Welchen Einfluss haben Medikamente auf das Blasenmanagement?	Behandlung	V	4,19	4	
22	Welche Faktoren (z.B. Dauer, Rehabilitationsverständnis) haben Einfluss auf die Qualität der rehabilitativen Versorgung von Menschen mit Querschnittlähmung?	Gesundheitsversorgungssystem	V	4,09	4	
23	Wie können Betroffene mit psychischen Krisen (z.B. Suizidalität, Sucht) besser behandelt werden?	Behandlung	V	4,06	4	
24	Können Menschen mit Querschnittslähmung gesellschaftlich teilhaben und ihr Leben selbständig gestalten?	Patientenfaktoren	B, V	4,34	3	
25	Welche Risikofaktoren für Dekubitus gibt es?	Gesundheitsproblem	V	4,26	3	
26	Wie können Harnwegsinfekte besser diagnostiziert werden?	Diagnose	V	4,17	3	
27	Wie kann Osteoporose behandelt werden?	Behandlung	A	3,98		
28	Wie kann die Öffentlichkeit besser über Querschnittlähmung und ihre Folgen aufgeklärt werden?	Öffentlichkeitsarbeit	V	3,94		
29	Kann die Blasenfunktion durch frühes Training erhalten bleiben?	Behandlung	B	3,88		
30	Welche Arbeitsbedingungen müssen geboten werden, um die Arbeit für Pflegende in der Paraplegiologie attraktiv zu machen?	Professionell Versorgende	V	3,83		
31	Wie sind die psychosozialen Kompetenzen von den an der Versorgung beteiligten Berufsgruppen ausgeprägt?	Professionell Versorgende	B, A	3,76		
32	Wie entwickelt sich das psychische Befinden nach der Erstrehabilitation?	Patientenfaktoren	V	3,72		
33	Wie können Menschen mit Querschnittslähmung besser mit Psychotherapie versorgt werden?	Gesundheitsversorgungssystem	V	3,69		
34	Welchen Einfluss haben Ausbildungsaspekte (z.B. Kommunikationstrainings) von Behandelnden auf ihren Umgang mit Menschen mit Querschnittlähmung?	Professionell Versorgende	V	3,66		

35	Wie kann ich als forschungsinteressierte*r Behandler*in, Angehörige*r und Betroffene*r besser an Studien mitarbeiten?	Gesundheitsversorgungssystem	B, V	3,52
36	Sind Kompressionsstrümpfe zur Thromboseprophylaxe wirksam?	Behandlung	V	3,35
37	Welchen Einfluss hat der Monatszyklus auf das Blasenmanagement?	Gesundheitsproblem	B	3,24
38	Kann eine Querschnittlähmung durch die Beeinflussung von Bewusstseinszuständen (z.B. Meditation, Hypnose) geheilt werden?	Behandlung	B	2,29

[1] B=Betroffene; A=Angehörige; V= professionell Versorgende

[2] Mittelwert aus der Bewertung anhand einer fünfstufigen Rating-Skala

[3] Häufigkeit der Auswahl bei 10 Möglichkeiten pro Teilnehmer*in

[4] Punkte nach Rangplatz (1. Rang = 10 Punkte … 10. Rang = 1 Punkt)
